# Predicting bacteriophage hosts based on sequences of annotated receptor-binding proteins

**DOI:** 10.1038/s41598-021-81063-4

**Published:** 2021-01-14

**Authors:** Dimitri Boeckaerts, Michiel Stock, Bjorn Criel, Hans Gerstmans, Bernard De Baets, Yves Briers

**Affiliations:** 1 KERMIT, Department of Data Analysis and Mathematical Modelling, Ghent University, Ghent, Belgium; 2 Laboratory of Applied Biotechnology, Department of Biotechnology, Ghent University, Ghent, Belgium; 3 Laboratory of Gene Technology, Department of Biosystems, KU Leuven, Leuven, Belgium; 4 MeBioS-Biosensors group, Department of BioSystems, KU Leuven, Leuven, Belgium

**Keywords:** Bacteriophages, Machine learning

## Abstract

Nowadays, bacteriophages are increasingly considered as an alternative treatment for a variety of bacterial infections in cases where classical antibiotics have become ineffective. However, characterizing the host specificity of phages remains a labor- and time-intensive process. In order to alleviate this burden, we have developed a new machine-learning-based pipeline to predict bacteriophage hosts based on annotated receptor-binding protein (RBP) sequence data. We focus on predicting bacterial hosts from the ESKAPE group, *Escherichia coli*, *Salmonella enterica* and *Clostridium difficile*. We compare the performance of our predictive model with that of the widely used Basic Local Alignment Search Tool (BLAST). Our best-performing predictive model reaches Precision-Recall Area Under the Curve (PR-AUC) scores between 73.6 and 93.8% for different levels of sequence similarity in the collected data. Our model reaches a performance comparable to that of BLASTp when sequence similarity in the data is high and starts outperforming BLASTp when sequence similarity drops below 75%. Therefore, our machine learning methods can be especially useful in settings in which sequence similarity to other known sequences is low. Predicting the hosts of novel metagenomic RBP sequences could extend our toolbox to tune the host spectrum of phages or phage tail-like bacteriocins by swapping RBPs.

## Introduction

Since their discovery, antibiotics have had an enormous positive impact on human health. Millions of patients suffering from bacterial infections have been successfully treated with antibiotics. However, today, bacterial resistance continues to increase due to overuse and misuse of antibiotics, resulting in selective pressure on bacterial communities^1^. Consequently, multidrug-resistant and even pandrug-resistant bacterial strains have appeared and are causing an increasing number of deaths worldwide. Broad-spectrum antibiotics represent the bulk of antibiotics approved for clinical use. Yet, they cause dysbiosis of the microbiome, negatively affecting the health-promoting effects of beneficial microbiota. Therefore, there is an increasing interest in narrow-spectrum antibiotics^2^. Both bacteriophages and phage tail-like bacteriocins (PTLBs, also called tailocins) are antibacterials with a narrow specificity that fulfill this need. Bacteriophages, or phages, are viruses that specifically infect and kill bacterial cells. Phages that reproduce via a lytic cycle disrupt the host cell wall at the end of the cycle, resulting in bacterial cell lysis^3^. PTLBs resemble tailed phages without the head^4^. They originate from defective domesticated prophages. In an altruistic system, they are produced by bacteria upon an SOS response and released by cell lysis to kill nutrient-competing bacteria, providing the surviving sister cells a competitive advantage^4–6^. Phages and PTLBs share the same primary determinant of specificity, i.e. one or more receptor-binding proteins (RBPs)^4,7–9^. RBPs recognize specific bacterial receptors on the cell surface such as polysaccharides (capsule, biofilm matrix, lipopolysaccharide), proteins, pili or flagella^5^. This initial recognition subsequently leads to infection of the bacterium by a phage or the depolarization of its membrane by a PTLB^10^.

Given their narrow specificity, phage therapy and PTLB application require a careful selection before treatment can take place. Typically, phages are isolated from their natural environments, after which their host spectrum is characterized experimentally^11^. Phages can also be selected and distributed from existing phage banks, a process which is increasingly being coordinated by global community platforms such as Phage Directory^12^. Determining the host range of phages can be done by infection tests, or by more recently developed methods such as microfluidic-PCR or PhageFISH^13–15^. Similarly, a PTLB with a matching specificity needs to be identified^6^. However, the isolation and characterization of phages and PTLBs can be a challenging and time-intensive process^6,16,17^.

Today, these challenges can be circumvented in new ways. Firstly, advances in metagenomics sequencing offer the possibility to directly discover new phage sequences from metagenomic contigs, avoiding the need to cultivate phages in the lab in order to discover new ones^18^. Over the past few years, a variety of tools such as MARVEL, Seeker and others have been built specifically for this purpose^19–24^. Secondly, engineering tools have been developed to modulate the host range of well-known phages and PTLBs by swapping or modifying their RBPs^5,6,25–27^. These tools demonstrate the potential of RBP engineering towards novel antibacterials with narrow and tunable host specificity.

Ideally, one has access to (cocktails of) engineered phages and PTLBs with a high diversity of RBPs targeting the broadest set of clinical strains and anticipating potential resistance development at the level of the bacterial receptor. More generally, we anticipate a future scenario in which more phage therapy efforts will have become personalized, driven by a platform that integrates artificial intelligence to help design therapeutic phages^28^. Recently developed tools enable the direct prediction of various specific phage protein classes, among which tail fiber proteins^29,30^. However, to this day, the lack of prior knowledge on the host specificity of these newly discovered RBPs still imposes a practical hurdle for engineering RBPs of phages and PTLBs towards novel antibacterials. As a result, there is a need for a separate tool that specifically bridges this last gap.

In this paper, we apply machine learning methods to predict bacterial hosts based on RBP sequence data. Firstly, with regard to clinical relevance, we construct a phage RBP database focused on hosts belonging to the ESKAPE organisms (an acronym standing for *Enterococcus faecium*, *Staphylococcus aureus*, *Klebsiella pneumoniae*, *Acinetobacter baumannii*, *Pseudomonas aeruginosa* and *Enterobacter* species^31^), supplemented with *E. coli*, *S. enterica* and *C. difficile*. Secondly, we represent raw DNA and protein sequences by a variety of numerical features that are used to train machine learning models. We then evaluate the predictive performance of four widely used machine learning methods: Linear Discriminant Analysis (LDA), Logistic Regression (LR), Random Forests (RF) and Gradient Boosting (GB). Finally, we compare the predictive performance of our best-performing model with results obtained by protein BLAST (BLASTp), the most widely used bioinformatics tool to study biological sequence data^32^. We show that our approach outperforms predictions by BLASTp when sequence similarity (measured as percentage identical residues) to other known sequences in the database drops below 75%.

## Results

### Construction of an RBP database focused on clinically relevant pathogens

**Figure 1:**
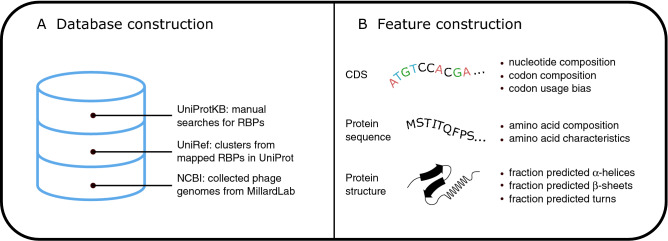
Graphical overview of database construction and feature construction. **(A) **Data were collected from three data sources: UniProtKB (via manual search), UniRef (via clusters that were mapped back to UniProtKB, May 2019) and collected phage genome data from MillardLab (www.millardlab.org, April 2019, downloaded via NCBI). **(B)** A total of 218 features (Supplementary Table S3) were constructed by collecting information from the coding DNA sequence (CDS), the protein sequence, and the protein structure.

Supplementary Table S1 summarizes the number of collected RBP sequences related to each of the bacterial hosts. In total, 1170 RBP sequences related to nine bacterial hosts were collected from three different public data sources. This collection was based both on key terms including ‘Tail fiber’, ‘Tail spike’ and variations thereof (e.g. ‘Long-tail fiber’) as well as on similarity-based clustering with UniRef. Within the *Enterobacter* genus, we only collected data for *Enterobacter cloacae*, the type species in the *Enterobacter* genus. We discarded identical RBP sequences prior to model construction. RBP sequences related to *E. coli* represent a large proportion of the database (n = 324), followed by sequences related to *K. pneumoniae* (n = 176) and *P. aeruginosa* (n = 117). RBP sequences related to *E. faecium* and *E. cloacae* are underrepresented (n = 4 and n = 29 before filtering, respectively) and were therefore excluded from subsequent analyses. Eventually, the final database consists of 887 RBP sequences. In this way, we trained our methods to discriminate between seven numerically encoded classes. Every class represents one of the bacterial host species: *S. aureus*, *K. pneumoniae*, *A. baumannii*, *P. aeruginosa, E. coli*, *S. enterica*, or *C. difficile*. Figure 1A depicts a visual overview of the database and its different data sources.

### Computation of identity percentages based on alignment and grouping of sequences

**Figure 2:**
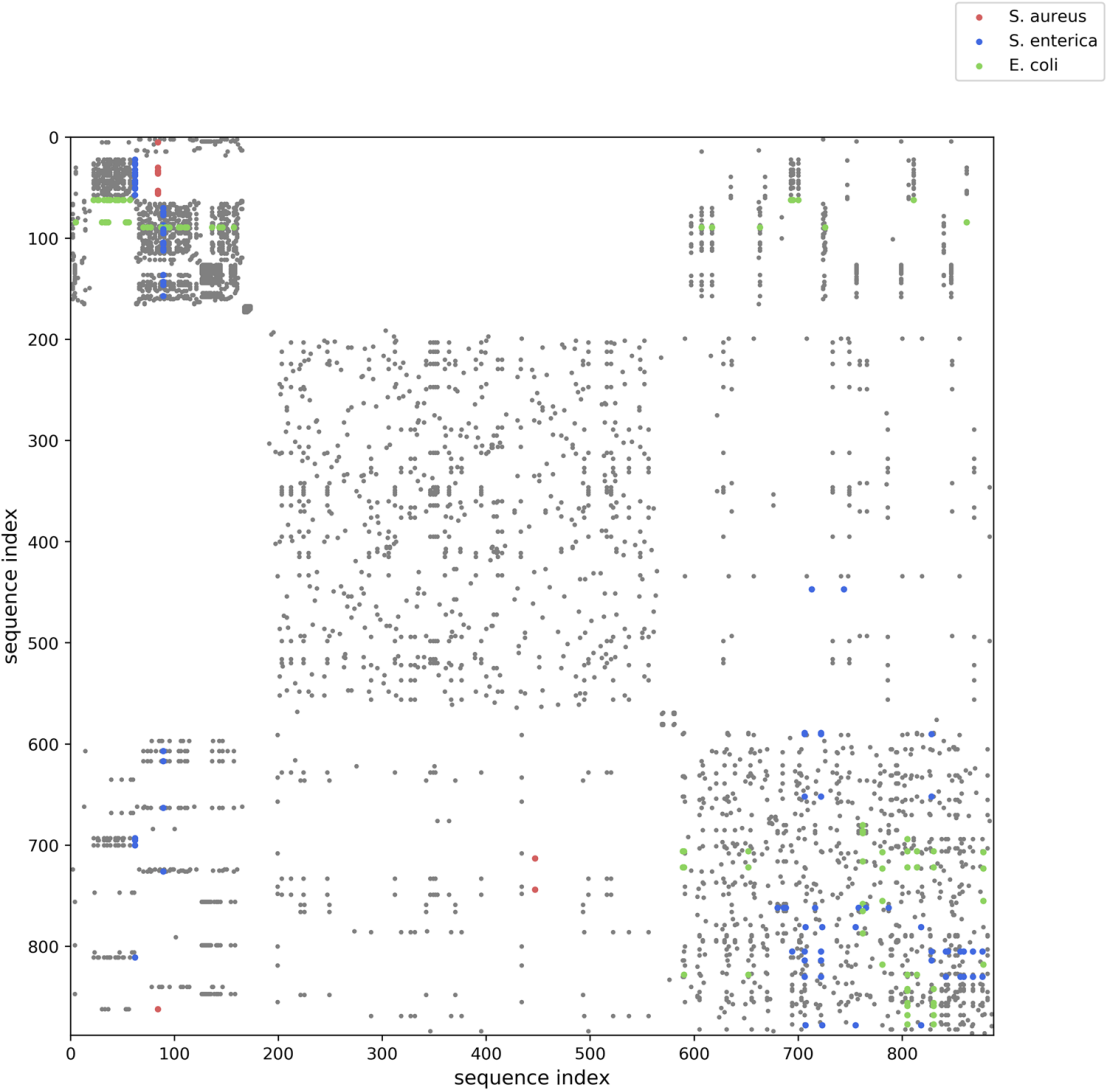
Symmetrical dot plot of sequence pairs in the database which are 95% or more identical to one another at the protein level. Local pairwise protein alignments were computed for all RBP sequences in the database using BioJulia (BioSequences version 1.1.0 and BioAlignments version version 0.3.0, https://biojulia.net). Afterwards, the identity percentage was computed for each pairwise alignment. Dots represent sequence pairs whose identity percentage is 95% or higher. Dots are colored in grey for sequence pairs that are related to the same bacterial host. Dots are colored in red, blue or green for sequence pairs that are related to different bacterial hosts. The sequence pair is colored according to the bacterial host related to the sequence index on the x-axis. Therefore, multiple colored dots with the same sequence index on the x-axis correspond to one particular sequence (same sequence index) with one particular host (same color). For example, at the top left, a single *S. enterica* RBP sequence (blue dots with the same sequence index on the x-axis) is related to multiple *E. coli* RBP sequences (green dots on the corresponding y-axis of the symmetrical plot).

To assess sequence similarities in the database, we computed identity percentages for pairwise local alignments between every two RBP sequences at the protein level. Figure 2 visually depicts the sequence pairs that are 95% or more identical (over the entire alignment) as a dot on a symmetrical dot plot. High sequence similarity in the database predominantly occurs between RBPs targeting the same bacterial species (grey dots). Additionally, sequence pairs exceeding the 95% threshold and whose related hosts differ, are colored according to the bacterial host related to the sequence index on the x-axis (colored dots). By observing the color of the corresponding dots on the y-axis (the plot is symmetrical), both related hosts for these sequence pairs can be identified. For example, at the top left of the plot, one *S. enterica* related RBP (the vertical line of blue dots with the same sequence index on the x-axis) is at least 95% identical to several *E. coli* related RBPs (green dots on the corresponding y-axis of the symmetrical plot). In addition, two *S. aureus* RBPs are significantly similar to *S. enterica* or *E. coli* phage RBPs.

Furthermore, RBP sequences from phages targeting the same host can also be highly dissimilar (Supplementary Table S2). For sequences related to *C. difficile*, the most dissimilar sequence was only 4.4% identical to other *C. difficile* RBP sequences. For sequences related to *S. aureus*, the most dissimilar sequence was 1.1% identical to other related sequences. For all other bacterial hosts, the most dissimilar sequence was less than 1% identical to other related sequences. These percentages were all computed from pairwise alignments as before. In summary, the database contains both highly similar and dissimilar RBP sequences related to the same host, as well as a few similar sequences related to different hosts. The presence of highly dissimilar RBP sequences suggests that the database includes RBPs targeting a large diversity of bacterial receptors.

### Selection of the most appropriate predictive model using F1 score and evaluation using precision-recall curves

Two linear and two nonlinear machine learning methods were selected to construct predictive models: Linear Discriminant Analysis (LDA), Logistic Regression (LR), Random Forests (RF) and Gradient Boosting (GB)^33–35^. Most machine learning models require numerical representations of the collected sequences as input during training. Therefore, in a first step, each of the 887 coding DNA sequences and its corresponding protein sequence in the database was represented by a vector of numerical values from which machine learning models can be trained. In total, each RBP was represented by a vector of 218 numerical features from the coding DNA sequence, the protein sequence and the protein structure (Fig. 1B, Supplementary Table S3). More specifically, 133 features were constructed from the coding DNA sequences (including nucleotide frequencies, GC-content, codon frequencies, and codon usage bias). Twenty features describe the relative abundance of amino acids. Fifteen other features describe physicochemical properties of the sequences (protein length, molecular weight, isoelectric point, aromaticity, and others). Three features describe the protein secondary structure in terms of the fractions of amino acids that are predicted to be present in an α-helix, β-sheet, or turn. Finally, 47 features describing protein sequences were implemented as described by Chen et al*.*, including composition, transition, and Z-scale features^36,37^.

**Figure 3:**
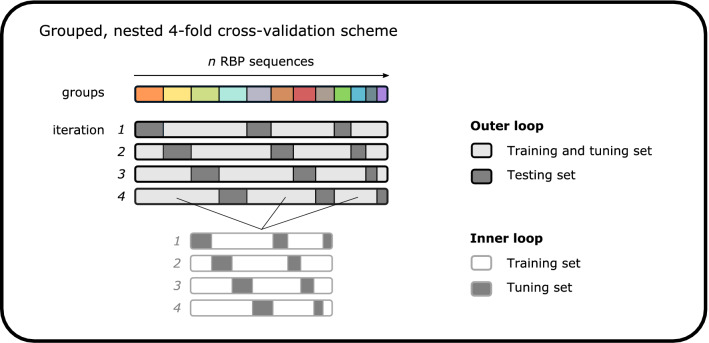
Grouped, nested fourfold cross-validation scheme to train and compare selected machine learning models. RBP sequences were grouped based on computed identity percentages between every sequence pair. In the inner loop, every model’s hyperparameters were optimized using grid search (with groups as defined before). In the outer loop, the performance of every optimized model was measured. In the outer loop as well, the defined groups control which sequences are in which fold.

**Figure 4:**
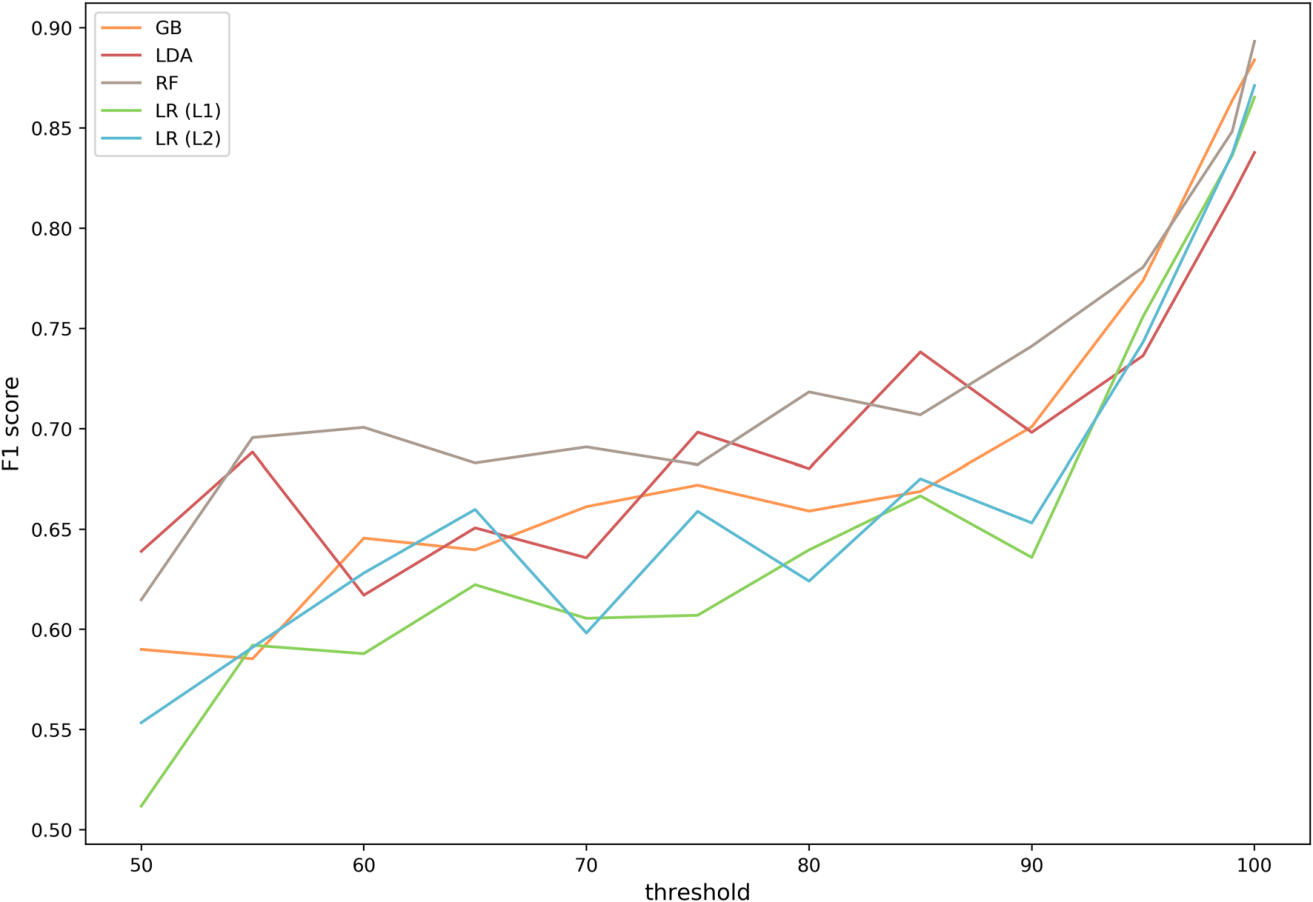
Cross-validated F1 scores of the tested predictive models across different thresholds for sequence similarity. Grouped, nested fourfold cross-validation was performed to tune the hyperparameters in the inner loop (GB, RF and LR) and measure performance in the outer loop. This was repeated for different thresholds of sequence similarity in the dataset that controlled the grouping in the cross-validation (i.e. the lower the threshold, the more sequences were grouped into the same fold making test set predictions more difficult). As performance metric, the F1 score was computed for every model at every threshold.

Secondly, model performance was evaluated through grouped, nested fourfold cross-validation (Fig. 3). We used cross-validation to reliably estimate performance by iteratively training models on different subsets (so-called folds) of the available data. In addition, the cross-validation was grouped. To cope with the high similarity (redundancy) of RBPs in training and evaluating predictive models, the computed identity percentages (amino acid level) were used to group sequence pairs that exceeded a set similarity threshold. Every sequence within a group always appeared in the same fold to prevent the models from overfitting. This grouping was repeated for thresholds on identity percentage ranging from 50 to 100%. The cross-validation was also nested, consisting of an inner loop and an outer loop. In the inner loop of cross-validation, the hyperparameters (i.e., the parameters that are set before model training begins) were optimized. In the outer loop, the F1 score (harmonic mean of precision and recall) was computed to assess the model performance. The model performance reaches 89.3% (F1 score, best-performing model) at the highest set threshold for sequence similarity and decreases to 51.1% (F1 score, worst-performing model) at the lowest set threshold (Fig. 4). As increasingly more sequences with a higher dissimilarity are grouped together in the same fold, sequences having an increasingly higher dissimilarity are left in the test sets. Therefore, generalizing on each test set becomes more difficult, and model performance decreases, as expected. However, taking sequence similarity into account in performance evaluation allows us to assess model overfit and deliver a realistic estimate of model performance for new RBP sequences (e.g., identified from metagenomics data) with a certain similarity to RBPs in the database. Overall, the RF model achieves the best performance over the different thresholds. At the lowest threshold of 50%, the RF model achieves an F1 score of 61.4%.

**Figure 5:**
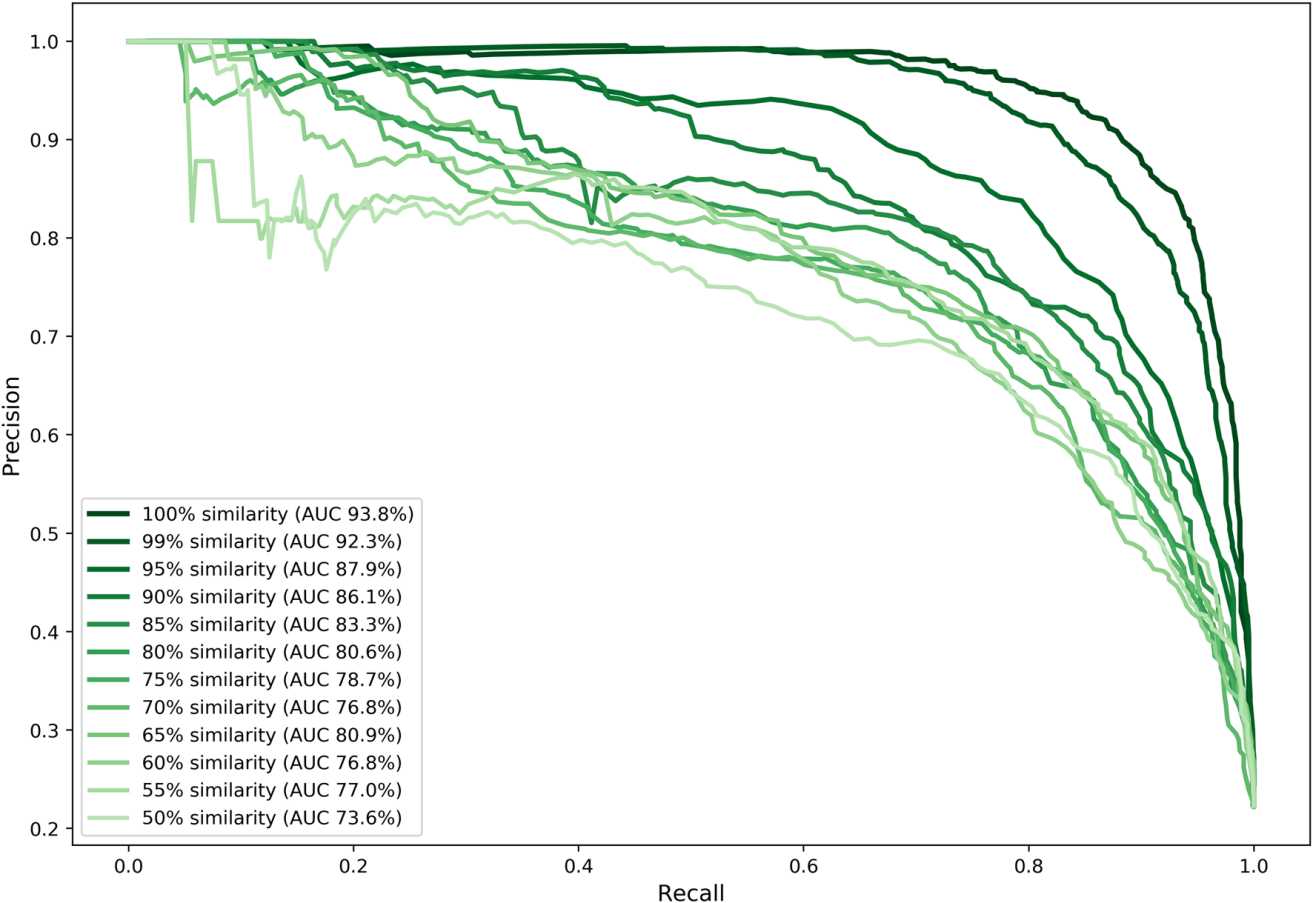
Cross-validated Precision-Recall (PR) curves and Area Under the Curve (AUC) of the best-performing predictive model (RF) across different thresholds for sequence similarity. Grouped, nested fourfold cross-validation was performed to tune the hyperparameters in the inner loop and compute weighted averaged precision and recall over all classes in the outer loop. This was repeated for different thresholds of sequence similarity in the dataset that controlled the grouping in the cross-validation (i.e. the lower the threshold, the more sequences were grouped into the same fold making test set predictions more difficult). In addition to plotting the PR curves, the AUC was computed as well (see legend).

The performance of the RF model was further examined by computing Precision-Recall (PR) curves and their corresponding Area Under the Curve (AUC). Precision is the proportion of positive predictions (i.e., related to a particular bacterial host) that are correct. Recall is the proportion of actual positives that are predicted correctly. The precision and recall of the model are evaluated across different thresholds on the output probabilities given by the model (not to be mistaken with the similarity thresholds). These output probabilities reflect the model's certainty in assigning each class (species) to an RBP sequence. The output probabilities over all classes sum to one. PR curves used here (as opposed to ROC curves) are especially useful in prediction problems with class imbalance, which is the case here. Again, PR curves were computed for similarity thresholds ranging from 50 to 100% (controlling the grouping of RBP sequences) in a grouped, nested fourfold cross-validation. Precision and recall were computed as weighted averages over all classes. Figure 5 shows the various PR curves at the different thresholds for sequence similarity. At a similarity threshold of 100%, the PR-AUC is 93.8%, decreasing to 73.6% at a similarity threshold of 50%. At both the highest and lowest threshold for sequence similarity, confusion tables for the RF model show that the accuracy of the prediction strongly varies by the bacterial host (Supplementary Table S4). At the highest threshold, the most accurately predicted bacterial host was *P. aeruginosa* (97.4% correct), while the least accurately predicted host is *A. baumannii* (76.6%). At the lowest threshold, the bacterial host that was most accurately predicted was *S. aureus* (92.9%). Conversely, the most difficult bacterial host to predict was *S. enterica* (30.4%). Wrongly classified *S. enterica* RBP sequences were most often predicted as being related to *E. coli* and *K. pneumoniae*. Analogously, actual *K. pneumoniae* RBP sequences were frequently misclassified as *E. coli* sequences. *S. enterica*, *K. pneumoniae* and *E. coli* all belong to the *Enterobacteriaceae*, which may explain why our RF model has difficulties making correct predictions at the lowest similarity threshold. It indicates that differentiation between those sequences takes place at a more subtle level that is not adequately reflected in the features.

### Biological interpretation of models and features

**Figure 6:**
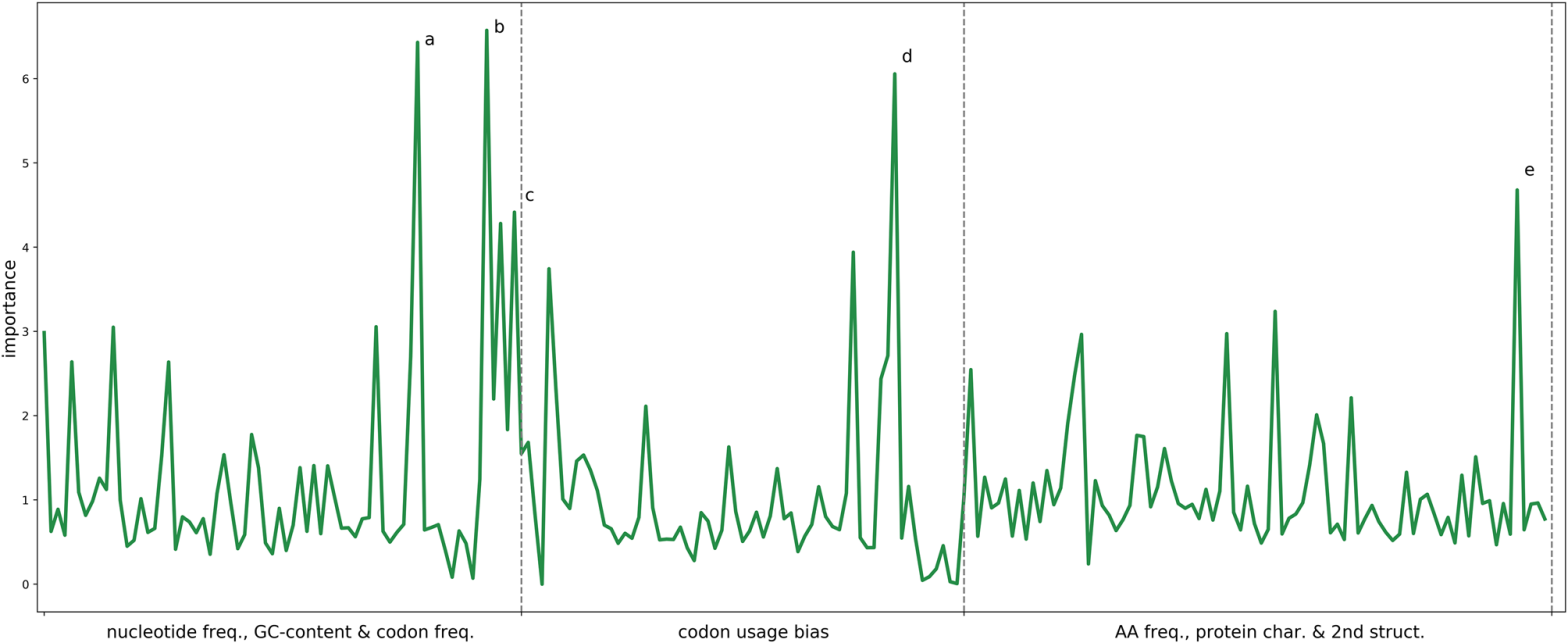
Plot of feature importance of a Random Forest (RF) model trained on all data with all features. An RF model was trained on all data with all features in Scikit-learn (version 0.22.1, https://scikit-learn.org/). The feature importance attribute was plotted. Five features influence predictions the most: **(a)** A nucleotide frequency, **(b)** TTA codon frequency, **(c)** TTA codon usage bias, **(d)** the first Z-scale descriptor and **(e)** GC content.

In order to assess which features contribute most to the predictions, feature importance was quantified using the feature importance attribute of an RF model trained based on all data without grouping (Fig. 6). The feature importance plot for the trained RF model (all features) shows that a variety of features across the different feature categories influence the predictions of the model. The top five features were: A nucleotide frequency, TTA codon frequency, TTA codon usage bias, the first Z-scale descriptor (lipophilicity of amino acids) and GC content. Interestingly, several features are (inversely) related to the presence or absence of G and C nucleotides (or GC content). Previously, an observed correlation between GC content of the phage genome and its host genome has been explained based on intense co-evolution^38^. However, further interpreting the biological relevance of these features is less straightforward. For example, the model output does not allow us to assess how (and what kind of) a change in TTA codon frequency would be associated with a potential change in host species. The first Z-scale descriptor is the only one among the most important features whose interpretation is not directly related to co-evolution of the GC-content between phage and host. This descriptor reflects the lipophilicity of the amino acids in the sequence^36^. Other important features deduced from the protein sequence and its physicochemical properties are the frequencies of the positively charged amino acids arginine and lysine, and specific transition features. Features related to the protein structure do not emerge as important.

Besides directly looking at features, we have separately trained our best-performing model based on the N-terminal part of each RBP sequence (defined as the first 200 AAs) and the C-terminal part of each sequence (defined as the entire sequence without its first 200 AAs and with a minimum length of 50 AAs). The N-terminal part is typically involved in attachment of the RBP to the phage tail, while the C-terminal part is involved in receptor binding. Structural N-terminal domains for attachment are commonly shorter than 200 amino acids^39^. PR curves were computed and compared to the previously computed PR curves of the best-performing model (trained on full sequences). Both training a model solely on the N-terminal part or C-terminal part results in a more substantial drop in performance as the threshold for similarity decreases (Figures S1 and S2). However, this drop in performance is less outspoken in the PR curve of our model trained on the C-terminal part, particularly at similarity thresholds above 80%.

### Comparison of a LOGOCV-trained RF model with BLAST

**Figure 7:**
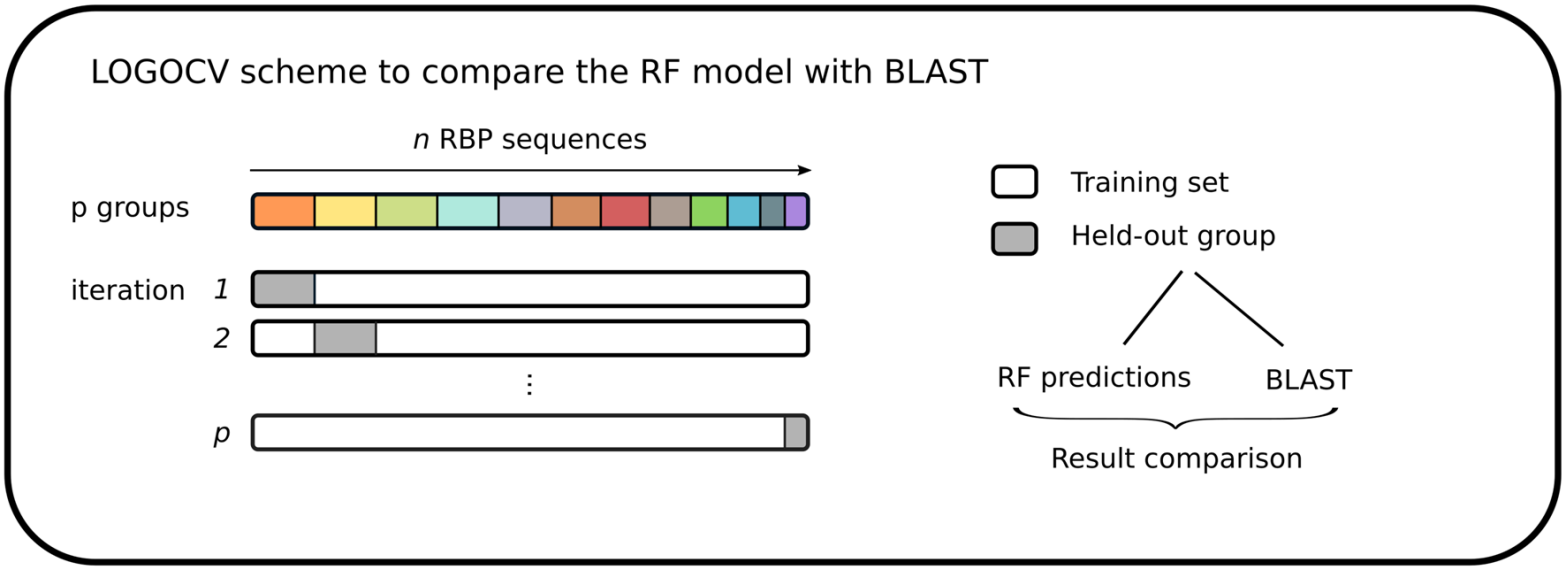
LOGOCV scheme used for the comparison of the final Random Forest (RF) model with BLASTp. An RF model is iteratively trained on all but one of *p* held-out groups. In every iteration, the trained model is used to make predictions for the held-out group. The sequences in the same held-out group are also subjected to a local BLASTp search against the database without the held-out group. Finally, both results are compared.

**Figure 8:**
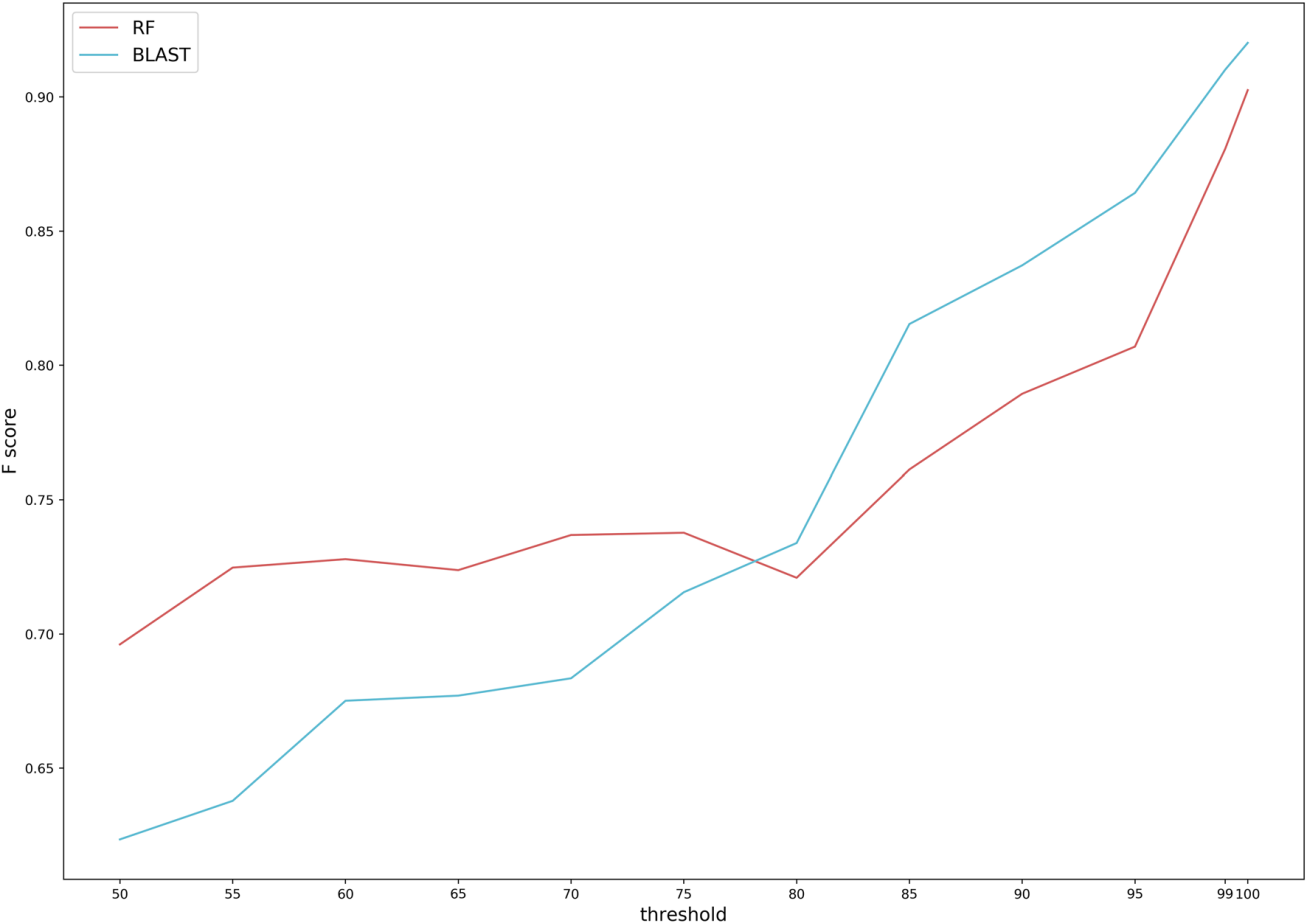
Computed F1 scores of the final Random Forest (RF) model and BLASTp in a Leave-One-Group-Out Cross-Validation (LOGOCV) across different thresholds for sequence similarity. Our final predictive model was compared with BLASTp using a LOGOCV scheme. In every round, a group was held out that was controlled by sequence similarity. The model was trained on all other sequences, after which the bacterial hosts related to the held-out group were predicted. The sequences in this group were also subjected to a local BLASTp search via BioPython against the database without the held-out group^36^. The LOGOCV was repeated for different thresholds of sequence similarity in the dataset that controlled the grouping in the cross-validation (i.e. the lower the threshold, the more sequences were grouped into the held-out group, leaving fewer sequences to train the model with or perform a BLASTp search against). At every threshold, the F1 score was computed for predictions made by the RF model and by BLASTp.

We have compared the predictive performance of our best-performing model with predictions obtained by BLASTp. First, the hyperparameters of the RF model were optimized as before based on grouped fourfold cross-validation. Subsequently, to further cope with the redundancy in the dataset and to compare both methods in a fair way, model training and predictions by both BLASTp and the RF model were carried out based on a leave-one-group-out cross-validation (LOGOCV) scheme (Fig. 7). In essence, an RF model with previously optimized hyperparameters was iteratively trained on all but one group of sequences (equivalent to the groups defined before) that was held out of the data. Subsequently, in every round of cross-validation, the bacterial hosts related to the held-out group were predicted. Each individual sequence in this group was also subjected to a local BLASTp (default parameters) search via BioPython^40^ against the database without the held-out group. The top hit with the lowest E-value in the database without the held-out group was selected as the prediction by BLASTp. Predictions by the RF model and BLAST were compared to the actual bacterial host. The F1 score was computed to compare the performance of both methods. This comparison was repeated for thresholds ranging from 50 to 100% sequence similarity that controlled the grouping of RBP sequences. As increasingly more sequences are grouped together (and thus are not available for the model to learn from or for BLASTp to search against), the performance (F1 score) of both methods decreases (Fig. 8). Moreover, at high thresholds (when highly similar sequences are available), our RF model reaches a comparable performance to BLASTp. More specifically, BLAST reaches an F1 score of 92.0% while our RF model reaches an F1 score of 90.3% at the highest threshold for sequence similarity (100%). In addition, for thresholds lower than 75% (meaning that sequences that are at least 75% identical are grouped together), our RF model outperforms BLAST. At the lowest threshold of 50%, the RF model reaches an F1 score of 69.6%, while BLAST reaches an F1 score of 62.3%.

Furthermore, confusion tables were constructed for predictions by the RF model and BLASTp at the lowest threshold for sequence similarity (Supplementary Table S5a and b for RF and BLASTp, respectively). Predictions by our RF model were most accurate for sequences related to *S. aureus* and *P. aeruginosa* (92.9% and 90.6%, respectively). Conversely, *S. enterica* and *A. baumannii* were the least accurately predicted classes (15.7% and 64.1%, respectively). Remarkably, some of the most accurately predicted classes by our RF model are among the least accurately predicted classes by BLASTp and vice versa. For example, *P. aeruginosa* was the least accurately predicted class by BLASTp (49.6%), while *K. pneumoniae* and *A. baumannii* were among the most accurately predicted classes by BLASTp (74.4% and 71.4%, respectively), but not by RF. Furthermore, the BLASTp method was unable to find a match for one of the *P. aeruginosa* RBP sequences. In other words, this sequence was too dissimilar to any of the other sequences in the database to find a hit via BLASTp. In contrast, this sequence was predicted correctly by our RF model.

## Discussion

Various computational tools have been proposed to predict phage host specificity. Traditionally, these tools have been based on whole phage genome sequences. Villaroel et al*.* developed HostPhinder, a tool that predicts a phage’s bacterial host based on genomic similarity by comparing *k*-mers^14^. Ahlgren et al*.* used alignment-free dissimilarity measures based on oligonucleotide frequency patterns between virus and host to predict the host of a given virus^41^. In another study, Galiez et al*.* predicted bacterial hosts in phage contigs using a homogeneous Markov model^42^. Finally, Leite et al*.* applied machine learning methods to predict interactions between phages and their bacterial host based on domain-domain interaction scores and protein primary structure information from both phage and bacterial host^43,44^. Noteworthy, none of the approaches mentioned above are explicitly focused on RBPs to predict host specificity. Recent advances in metagenomics and the availability of tools to directly predict phage RBPs from metagenomic datasets enable the discovery of many previously unknown RBPs^30^. Coupled with advances in synthetic biology to switch host specificity by transplanting RBPs or domains thereof in a well-known phage scaffold, these tools further support RBP engineering efforts harnessing this potentially wide variety of novel phage RBPs that are predicted from metagenomic datasets^25–27^. However, a tool that predicts host specificity from RBPs is still lacking. Therefore, in this study, we developed a new approach to predict host specificity at the species level, based on a machine learning model and RBP sequence data. More specifically, our model predicts bacterial hosts belonging to *S. aureus*, *K. pneumoniae*, *A. baumannii*, *P. aeruginosa*, *E. coli*, *S. enterica* and *C. difficile* based on information from phage RBP sequences. Pairwise alignments between the sequences indicated that high similarity is present among the RBP sequences in the database. On the one hand, this is a consequence of automatic annotation of sequence data, which is a common practice in many of the biological sequence databases (i.e., similar sequences get annotated in similar ways). In addition, sequences were recruited based on sequence alignments. On the other hand, phages are known to intensively recycle and exchange (parts of) their RBPs by horizontal transfer^39^. To measure performance in a realistic manner (and to avoid overfitting), we have implemented a grouped cross-validation strategy that excludes highly similar sequences in the same fold, based on the computed pairwise alignments. The higher the number of similar sequences that are grouped together (and thus excluded from the same fold), the more difficulty models have in generalizing to new, unseen sequences. In this regard, more diverse RBP sequences related to the species mentioned above can benefit model performance, especially when making predictions for sequences that are very dissimilar to any of the other known sequences.

Our results show that machine learning models provide opportunities for accurate in silico prediction of host specificity based on RBP sequences. Our final predictive model starts outperforming predictions by BLASTp when sequence similarity (measured as percentage identical residues at the amino acid level) to other known sequences in the database drops below 75%. Above this threshold, the performance of our model increases and is comparable to that of BLASTp. Expectedly, BLASTp’s performance decreases substantially when sequence similarity in the database decreases because it is based on direct comparison between sequences. In practice, an expert might be capable of making more accurate predictions by looking at more than just the top BLASTp hit. However, the performance of our machine learning tool also decreases (though less substantially) when similarity drops, as our tool predicts patterns that are less familiar to the ones it has seen in the training phase. Together, this indicates that predictive models can be a complementary tool to BLASTp and are especially useful in settings in which sequence similarity to other known sequences in the database is low. Metagenomic datasets are a typical setting in which low sequence similarity to known sequences can occur. For example, as few as 5% of the viruses in the human skin microbiome are similar to already known viruses^45^. Indeed, previously Fernández-Ruiz et al. have discovered a large number of new endolysins from previously uncultured phages^46^. Many of these endolysins showed novel domain architectures. In the same way, we expect metagenomic datasets to contain many RBPs that are dissimilar to already known RBPs. Additionally, the most accurately predicted classes by our model are the least accurately predicted classes by BLASTp and vice versa. One explanation for this behavior is that the underlying co-evolutionary signal might be more or less conserved depending on the class, compared to the information in an alignment. Again, this suggests that BLASTp and our predictive model can be deployed in a complementary manner to maximize correct predictions. Furthermore, we chose to train and evaluate our final predictive model based on LOGOCV to compare it with BLASTp. This is the best approximation to a realistic scenario in which the model is trained on all available data and later makes predictions for new, unseen data. The BLASTp comparison was executed against our RBP database to ensure an appropriate comparison of both methods. This does not necessarily reflect a practical setting in which a scientist would perform a BLASTp analysis of a newly discovered RBP against the NCBI database and consider more than only the top hit (given the modular nature of RBPs). Comparing such an approach with our model predictions would be less straightforward because multiple top predictions made by BLASTp cannot be interpreted probabilistically. In addition, we repeated our comparison for multiple thresholds of sequence similarity, providing an estimation of how performant our model will be in different scenarios of making predictions for novel RBPs, including RBPs with high dissimilarity to other RBPs. However, fully assessing the generalizability of the model remains difficult. In particular, the limited diversity of the database for classes with a limited number of instances, such as *C. difficile* or *A. baumannii*, makes it difficult to predict how well the machine learning model would generalize to new sequences related to these classes. More data, especially for the classes that are underrepresented, can only improve real-world performance of the predictive model. In this regard, automation of the data acquisition process can further expand the database in the future. Additionally, many RBPs target species other than the species we trained our predictive model on. Indeed, we have strategically focused on the most problematic bacterial pathogens with regard to antibiotic resistance. RBPs that are not related to any of these species should result in low, equally distributed probability scores across the different classes (i.e., the model is not certain about any of the classes). However, it would be equally interesting to incorporate RBPs from other bacterial species in the training of our predictive models.

In our approach, we deliberately focus on predictive models that are trained based on RBP sequences as predictors. We show that by only considering the RBP, accurate predictions for its related bacterial host can be made at the species level, without having to consider the entire phage genome or interacting bacterial genome. We envision our tool being used for applications in the field of RBP engineering of phages and PTLBs. More specifically, our tool is useful to predict the host specificity of novel RBPs that were discovered in metagenomic datasets. Incorporating these novel RBPs into therapeutic phages or PTLBs could significantly increase the diversity of receptors targeted by these antibacterials.

Although phage RBPs constitute the primary determinant of phage host specificity, these proteins are not the only determinant. Indeed, other factors influencing specificity include restriction-modification systems, CRISPR-Cas immunity and superinfection exclusion by prophages, among others^47,48^. These factors further determine host specificity at the strain level. An insufficient number of known strain-level interactions related to clinically relevant bacterial strains currently hinders predictions at the strain level as well as incorporating bacterial genomic information into our models. In phage RBP engineering, these additional mechanisms of resistance may impede infection by phages with a swapped RBP. However, with regard to PTLB engineering, this is not a limitation, as PTLB specificity strictly depends on the RBP^5^. In addition, predictions at the species level are still useful to separate the few potentially interesting RBPs from the many that are not worth further investigation and to compose cocktails that cover various strains of the same species.

Furthermore, some phages possess multiple RBPs (e.g., central tail spikes, (branched) side tail fibers, long and short tail fibers or distal tail proteins with carbohydrate-binding modules) that influence specificity^39,49^. Phages may have multiple RBPs to expand the host spectrum. For example, *E. coli* phage K1-5 has a dual RBP system targeting both the K1 and K5 capsule. Other examples can be found among *Klebsiella* phages including phage ΦK64-1, which has eleven RBPs targeting at least ten different capsular serotypes^39,50^. Other phages have two RBPs to bind to a primary and secondary receptor, respectively. For example, *E. coli* phage T5 reversibly binds with its L-shaped side tail fiber (T5pb9) to the polymannose O-antigen^51^, while the tail tip (T5pb5) protein is critical for recognition of the outer membrane ferrichrome transporter FhuA and for infection^52^. Due to a general lack of detailed sequence annotation, our database comprises all types of RBPs. Our approach is therefore not able to discern between these different types of RBPs.

Different features with high importance in the final model directly or indirectly relate to GC content and may thus originate from the intense co-evolution between the phage and host genome. Our results show that capturing this information from a single gene instead of from a complete genome is already sufficient to obtain a good prediction model. Yet, (parts of) RBP genes are also highly prone to horizontal transfer^39^. In the case of a recent horizontal gene transfer, the specificity of an RBP may therefore be harder to predict on the basis of these features. Sequence characteristics other than those related to GC content also have a determining effect on the prediction of the host specificity (e.g., secondary structure elements). In general, these characteristics can be more informative and of practical use in RBP engineering efforts. However, our current feature representation does not adequately incorporate a more diverse set of sequence characteristics.

Additionally, we have compared the performance of our best-performing model when it was trained on either the N-terminal or C-terminal end of each RBP sequence (Figures S1 and S2). Generally, the N-terminal RBP domains (< 200 AAs) are more conserved and involved in attachment of the tail fiber to the phage tail, while the C-terminus is highly variable and involved in receptor binding^39^. The performance (measured as AUC) decreases compared to predictions for the full length RBP sequences, but less rapidly for predictions based on the C-terminal end, at least for higher thresholds of sequence similarity. The majority of the sequences contain a C-terminal part that is longer than the N-terminal part. Longer sequences will better reflect the co-evolutionary signal of the entire genome, which can equally explain why the C-terminal parts are more predictive. Overall, these results provide further evidence for the co-evolutionary signal that is represented by our features. Thus, our model predictions are more accurate when considering the entire RBP sequence. However, with regard to swapping RBPs in phage scaffolds, only the C-terminal receptor-binding domain will be exchanged, while the N-terminal anchor domain of the accepting phage scaffold remains in place for functional attachment of the swapped C-terminus to the phage tail. Therefore, additional characterization of each suitable RBP (after host prediction) and appropriate delineation of its C-terminal receptor-binding domain are required to substitute the C-terminal receptor-binding domain of the accepting phage scaffold^25^.

To conclude, with an ESKAPE-focused RBP database and an accurate machine learning model, more RBP engineering efforts in phages and PTLBs can be undertaken to target the clinically relevant ESKAPE pathogens and speed up the development of narrow-spectrum phage-based solutions against these increasingly resistant bacterial pathogens.

## Methods

### Database construction

We have collected raw RBP sequence data from three publicly available data sources (Fig. 1A). Firstly, UniProtKB was queried for ‘phage tail fiber’, a term often used to describe RBPs^53^. Queries were restricted to the Caudovirales group of viruses to enhance the relevance of the results. Secondly, UniprotKB was queried again, now with the genus of bacterial hosts of interest appended with ‘phage tail fiber’. Often, phages are named after the genus of the bacterial host they infect. The resulting protein sequences of this second query were mapped to UniRef50 to form clusters with the other proteins in UniProtKB. Each of the other proteins in UniProtKB that is at least 50% identical and overlaps with at least 80% of the seed sequence was placed in the same cluster^54^. The UniRef50 clusters containing a phage RBP as representative sequence (i.e., the best-annotated sequence in each cluster) were selected. These clusters were subsequently mapped back to UniProtKB to download the protein sequence data from that cluster. In total, sequence data from 129 unique clusters related to the ESKAPE organisms, *E. coli*, *S. enterica*, and *C. difficile* were downloaded (May, 2019). Subsequently, the protein sequence, EMBL identifier, taxonomic identifier, protein name, and organism name were collected from these data. Further processing was done using BioPython^40^. This processing included accessing NCBI to collect the host name and coding DNA sequence related to every sequence collected from UniProtKB^55^. Sequences of which the host name was not among the ESKAPE organisms, *E. coli*, *S. enterica*, and *C. difficile* were discarded. The third source of raw data collection was the phage genome database constructed by Millard and colleagues. A total of 11914 phage genome accession numbers were downloaded from their website (www.millardlab.org, April 2019). NCBI was accessed to download the sequence records for every phage genome through its genomic identifier. The downloaded NCBI record of every phage genome that is related to an ESKAPE organism, *E. coli*, *S. enterica* or *C. difficile* was searched for annotated RBP proteins based on key terms including ‘Tail fiber’, ‘tail fiber’, ‘Tailfiber’, ‘tailfiber’, ‘Long-tail fiber’, ‘Tail spike’, ‘tail spike’, ‘Tailspike’ and ‘tailspike’. The coding DNA sequence and protein sequence of every annotated RBP were collected, together with the protein name, the host name, the organism name, the EMBL identifier, and the taxonomic identifier.

Finally, three filters were applied to the entire database. Firstly, sequences with undetermined amino acids (X) or undetermined nucleotides (N) were removed from the database. Secondly, sequences that were shorter than 200 amino acids were also removed to avoid predictions based on a single protein domain rather than an entire protein sequence. Thirdly, sequences with annotations that are unrelated to RBPs (including hinge connectors, portal proteins, assembly proteins, short-chain dehydrogenases and RNA ligases) were also discarded from the database.

The number of sequences related to each of the bacterial hosts varied widely (Supplementary Table S1). We have discarded sequences related to *E. faecium* and *E. cloacae* from further analyses due to the low number of collected sequences for these two species. Additionally, identical RBP sequences were also discarded prior to model construction. The final database consisted of 887 RBPs related to seven bacterial hosts. Every processing step was automated using the Python programming language.

### Construction of a feature representation

Every coding DNA sequence and corresponding protein sequence in the database was represented by a vector of numerical values on the basis of which machine learning methods can learn patterns during a process called training. Our goal was to represent every sequence with many different characteristics that describe the sequence and reflect the diversity between sequences.

In total, every RBP was represented by a vector of 218 numerical features extracted from the coding DNA sequence, the protein sequence and the protein structure (Fig. 1B, Supplementary Table S3). First, 133 features were constructed from the coding DNA sequences. These features include nucleotide frequencies, GC-content, codon frequencies, and codon usage bias. Codon usage bias was computed by counting the occurrence of each codon and subsequently dividing by the total number of counts from synonymous codons (i.e., codons that correspond to the same amino acid). Furthermore, features were also constructed based on the primary protein sequence. More specifically, 20 features describe the relative abundance of amino acids. Fifteen other features describe various physicochemical properties of the sequences. These include protein length, molecular weight, isoelectric point, aromaticity, and others. Additionally, three features describe the protein secondary structure in terms of the fractions of amino acids that are predicted to be present in an α-helix, β-sheet, or turn. Finally, 47 features describing protein sequences were implemented as described by Chen et al*.*, including composition, transition, and Z-scale features^36,37^. The composition and transition features represent the overall composition and transition of several amino acid attributes, including relative hydrophobicity, the predicted secondary structure and the predicted solvent exposure^56^. The Z-scale encodes every amino acid by five principal properties describing chemical and proton NMR spectroscopy parameters^36,37^.

### Model construction and performance validation

The seven remaining bacterial hosts (besides *E. faecium* and *E. cloacae* of which the sequences were discarded, see earlier) were numerically encoded as seven classes in the database. The predicted output for every remaining sequence in the database was one of these seven numerically encoded classes. In this way, our trained models performed a seven-class classification, in which every class represents one of the bacterial host species.

Two widely used linear and two nonlinear machine learning methods were selected to construct predictive models. The two linear methods were Linear Discriminant Analysis (LDA) and Logistic Regression (LR)^33^. LDA is a simple linear generative method that models the different classes as multivariate normal distributions with an identical covariate structure. Because this covariance structure is shared across different classes, it can cope with having few examples in certain classes. In contrast, LR is a linear discriminative method for classification. The LDA method used the Least-Squares QR (LSQR) method for optimization ^57^. An LR model was fitted both with an L1-regularization and an L2-regularization and used the Stochastic Average Gradient Augmented (SAGA) optimization method^58^. The nonlinear methods were Random Forests (RF) and Gradient Boosting (GB)^34,35^. Both RF and GB are ensemble methods that use collections of decision trees to obtain a stronger model. In RF, these trees are trained independently using bootstrap aggregation (sampling with replacement followed by a majority vote). GB, in contrast, fits the trees sequentially: each new tree is adapted to improve the performance of the current ensemble. Many machine learning practitioners consider RF and GB among the top off-the-shelf machine learning methods for unstructured data^59^. For the LR and RF methods, class weights were balanced. This option was not available for the LDA and GB methods. For every model, we used the implementation available in the Scikit-learn package in Python^60^.

We have measured model performance based on nested, grouped fourfold cross-validation (Fig. 3). Cross-validation amounts to training models iteratively on different subsets of the available data. First, local pairwise alignments were computed between every RBP sequence to assess redundancy in the database. These pairwise alignments were computed using BioJulia. The resulting identity scores were used to group the RBP sequences in order to carry out a grouped fourfold cross-validation. Sequence pairs that exceeded a set threshold for identity percentage were grouped together. In this way, various groups of sequences were constructed in which no identity percentage between a sequence from one group and a sequence from another group exceeded the threshold. Therefore, training and evaluation was based on all available data, while ensuring that similar sequences (as measured by identity percentage exceeding the threshold) always occurred in the same split (or fold) of the nested cross-validation. This grouping was repeated for thresholds ranging from 50 to 100% in steps of 5%, and additionally for 99%. For every grouping, predictive models were trained and evaluated in the following manner. Firstly, the feature representation for all RBP sequences was standardized to have zero mean and unit variance. Secondly, the different methods listed above were used to construct predictive models. The values of every model’s hyperparameters were optimized in the inner loop of the cross-validation scheme in a grid search (except for LDA, which has no hyperparameters). The different hyperparameters that were optimized for every model are reported in Supplementary Table S6. For the LR model, the complexity parameter C was optimized. This parameter controls the regularization to prevent overfitting. For the RF and GB models, the number of trees in the model was optimized. In general, a higher number of trees leads to better performance but increases the time needed to train the model. Additionally, the number of features to consider when looking for the best split was optimized for the RF model. A higher number for this parameter increases the predictive power of individual trees, but also increases the correlation between the individual trees. Every hyperparameter value was optimized using accuracy as a performance measure (in the inner loop of cross-validation). Finally, the performance of each model was tested based on unseen data in the outer loop of the cross-validation scheme. Here, the F1 score was computed and used to quantitatively compare the different predictive models (for every set threshold on identity percentage) and to identify the best-performing model. The F1 score is defined as the harmonic mean of precision and recall. These three metrics were computed as weighted averages over all classes. Furthermore, the best-performing model was analyzed in more detail by computing precision-recall curves. These curves were also computed for thresholds ranging from 50 to 100% (controlling the grouping of RBP sequences) in a grouped, nested fourfold cross-validation. Here as well, precision and recall were computed as weighted averages over all classes. Finally, a confusion table was constructed for predictions by the best-performing model at the lowest threshold for sequence similarity.

### Assessment of feature importance

An RF model was chosen as the final predictive model based on the F1 score. The relevance of the DNA and protein features was examined by quantifying feature importance using the feature importance attribute of the RF model, after training the RF model based on all data and all features with standard settings for hyperparameters in Scikit-learn^60^. In addition, precision-recall curves were computed using the best-performing model based on the N-terminal part of the sequence (defined as the first 200 amino acids) or the C-terminal part of the sequence (defined as the entire sequence without its first 200 amino acids and with a minimum length of 50 amino acids). These precision-recall curves were subsequently compared to the previously computed precision-recall curves of the best-performing model trained on full sequences (see previous section).

### Comparison of the machine learning approach with BLASTp

The hyperparameters of the RF model were optimized based on grouped fourfold cross-validation. The optimized hyperparameters were then used to train the final predictive model based on a leave-one-group-out cross-validation (LOGOCV) scheme (Fig. 7). Here, the RF model was iteratively trained on all but one group in the database. In every round of cross-validation, the bacterial hosts related to the held-out group were predicted. The same group that was held-out was also used to perform a local BLASTp search via BioPython against the database without the held-out group^40^. Each individual sequence in a held-out group was subjected to a BLASTp search. Default parameters were used and the top hit (lowest E-value) in the database (without the held-out group) was selected as the prediction by BLASTp. Again, this comparison was repeated for thresholds ranging from 50 to 100% in steps of 5%, and additionally for 99%, controlling the grouping of RBP sequences. In this way, the effect of similar RBP sequences present in the database was examined for the predictive model and BLASTp at the same time. Predictions by the predictive model and BLASTp were compared to the actual bacterial host for every threshold of identity percentage. As before, the F1 score was computed as a metric for model performance and to compare against predictions by BLASTp. Here as well, the F1 score was computed as a weighted average over all classes. Furthermore, confusion tables were constructed for predictions by BLASTp and the RF model at the lowest threshold for sequence similarity to compare both methods in more detail.

## Supplementary Information

Supplementary Information.

## Data Availability

The constructed database and code for the analyses are available on GitHub at https://github.com/dimiboeckaerts/BacteriophageHostPrediction.
