# Supplementary material for: Predicting bacteriophage hosts based on sequences of annotated receptor-binding proteins
Source: Sci Rep. 2021 Jan 14;11:1467. doi: 10.1038/s41598-021-81063-4 (PMC7809048; doi:10.1038/s41598-021-81063-4)
Supplement: Supplementary file 1 — Supplementary Information. [file 41598_2021_81063_MOESM1_ESM.pdf]

Article

Predicting bacteriophage hosts based on sequences of annotated receptor-binding proteins

Dimitri Boeckaerts[1,2], Michiel Stock[1], Bjorn Criel[2], Hans Gerstmans[2,3,4], Bernard De Baets[1], Yves Briers[2,*]

[1]KERMIT, Department of Data Analysis and Mathematical Modelling, Ghent University, Belgium

[2]Laboratory of Applied Biotechnology, Department of Biotechnology, Ghent University, Belgium

[3]Laboratory of Gene Technology, Department of Biosystems, KU Leuven, Belgium

[4]MeBioS-Biosensors group, Department of Biosystems, KU Leuven, Belgium

*Corresponding author: Yves.Briers@UGent.be

Supplementary Information

Supplementary Table S1: Number of collected RBP sequences. Overview of the number of collected RBP sequences related to every bacterial host and the proportion of every bacterial host's occurrence.

Bacterial host group	Number of RBP sequences	Number of RBP sequences after filtering	Proportion of final database
Enterococcus faecium	4	0	0%
Staphylococcus aureus	96	70	7.9%
Klebsiella pneumoniae	227	176	19.8%
Acinetobacter baumannii	69	64	7.2%
Pseudomonas aeruginosa	156	117	13.2%
Enterobacter cloacae	29	0	0%
Escherichia coli	399	324	36.5%
Salmonella enterica	148	115	13.0%
Clostridium difficile	42	21	2.4%
**Total**	**1170**	**887**	**100%**

Supplementary Table S2: Identity percentage (amino acid level) of the most dissimilar RBP sequence pair within the same group of a related host (bacterial species). Overview of the identity percentage of the most dissimilar RBP sequence pair within each related host group (i.e. sequences that are both related to the same host). Identity percentages were computed after performing pairwise local alignments in BioJulia.

**Bacterial host group**	**Identity percentage of the most dissimilar sequence pair within the same group**
Staphylococcus aureus	1.09%
Klebsiella pneumoniae	0.70%
Acinetobacter baumannii	0.78%
Pseudomonas aeruginosa	0.82%
Escherichia coli	0.62%
Salmonella enterica	0.88%
Clostridium difficile	4.46%

Supplementary Table S3: Computed features from coding DNA sequences (a) and protein sequences (b). In total, 218 features describing the coding DNA sequences and protein sequence were computed. The features computed from the coding DNA sequences were nucleotide frequencies, GC-content, codon frequencies, and codon usage bias. The features computed from protein sequences were amino acid (AA) frequency, 15 physicochemical properties, 3 features describing predicted secondary structure, and 47 features describing protein composition, protein transition and Z-scale.

(a) Features computed from coding DNA sequence

**Description**	**Number of features**	**Reference**
Nucleotide frequency	4	/
GC-content	1	Zhou & Liu, 2008
Codon frequency	64	Sastry *et al.*, 2017
Codon usage bias	64	Roux *et al.*, 2015

(b) Features computed from protein sequence

**Description**	**Number of features**	**Reference**
AA frequency	20	Al-Shabib *et al.*, 2007
Molecular weight	1	Al-Shabib *et al.*, 2007
Protein Length	1	/
Iso-electric point	1	Sastry *et al.*, 2017
Aromaticity	1	Sastry *et al.*, 2017
Instability	1	Sastry *et al.*, 2017
Flexibility	1	Sastry *et al.*, 2017
Aliphatic AA fraction	1	Sastry *et al.*, 2017
Uncharged polar AA fraction	1	Sastry *et al.*, 2017
Polar AA fraction	1	Sastry *et al.*, 2017
Hydrophobic AA fraction	1	Sastry *et al.*, 2017
Positively charged AA fraction	1	Sastry *et al.*, 2017
Negatively charged AA fraction	1	Sastry *et al.*, 2017
Sulfur containing AA fraction	1	Sastry *et al.*, 2017
Amide containing AA fraction	1	Sastry *et al.*, 2017
Alcohol containing AA fraction	1	Sastry *et al.*, 2017
Fraction of AAs in α-helix	1	Sastry *et al.*, 2017
Fraction of AAs in β-sheet	1	Sastry *et al.*, 2017
Fraction of AAs in turn	1	Sastry *et al.*, 2017
Protein composition	3	Chen *et al.*, 2018
Protein transition	39	Chen *et al.*, 2018
Protein Z-scale	5	Chen *et al.*, 2018

Supplementary Table S4: Confusion tables of predictions by the Random Forest (RF) model in a grouped, nested 4-fold cross-validation at the highest threshold (100%) and at the lowest threshold (50%) for sequence similarity. Summary of the number of predicted bacterial hosts versus actual bacterial hosts for the RF predictive model based on grouped, nested 4-fold cross-validation at the highest and lowest threshold (100% and 50%, respectively) of sequence similarity that controls the grouping of sequences in the cross-validation. Rows represent the actual bacterial hosts, whereas columns represent the predicted bacterial hosts.

a) highest threshold of sequence similarity (100%)

		Predicted bacterial hosts						
		S. aureus	***K. pneumoniae***	***A. baumannii***	***P. aeruginosa***	***E. coli***	***S. enterica***	***C. difficile***
Actual bacterial hosts	***S. aureus***	66	0	0	0	3	1	0
	K. pneumoniae	0	137	0	3	33	3	0
	A. baumannii	1	2	49	2	10	0	0
	P. aeruginosa	0	1	1	114	1	0	0
	E. coli	1	7	1	0	310	5	0
	S. enterica	0	3	0	0	14	98	0
	C. difficile	3	0	0	0	0	0	18

b) lowest threshold of sequence similarity (50%)

		Predicted bacterial hosts						
		S. aureus	***K. pneumoniae***	***A. baumannii***	***P. aeruginosa***	***E. coli***	***S. enterica***	***C. difficile***
Actual bacterial hosts	***S. aureus***	65	0	1	0	2	0	2
	K. pneumoniae	0	126	0	4	42	4	0
	A. baumannii	0	2	35	3	24	0	0
	P. aeruginosa	0	8	1	103	5	0	0
	E. coli	0	95	11	2	213	3	0
	S. enterica	0	35	4	0	41	35	0
	C. difficile	2	0	0	0	0	0	19

Supplementary Table S5: Confusion table of predictions by the Random Forest (RF) model and BLAST in a LOGOCV at the lowest threshold (50%) for sequence similarity. Summary of the number of predicted bacterial hosts versus actual bacterial hosts for the RF predictive model and BLAST based on leave-one-group-out cross-validation at the lowest threshold (50%) of sequence similarity that controls the grouping of sequences in the cross-validation. Rows represent the actual bacterial hosts, whereas columns represent the predicted bacterial hosts.

a) Confusion table of predictions by the RF model

		Predicted bacterial hosts						
		S. aureus	***K. pneumoniae***	***A. baumannii***	***P. aeruginosa***	***E. coli***	***S. enterica***	***C. difficile***
Actual bacterial hosts	***S. aureus***	65	0	0	0	3	0	2
	K. pneumoniae	0	118	0	3	54	1	0
	A. baumannii	0	1	41	3	19	0	0
	P. aeruginosa	0	5	1	106	5	0	0
	E. coli	0	44	4	1	273	2	0
	S. enterica	0	47	0	0	50	18	0
	C. difficile	5	0	0	0	0	0	18

b) Confusion table of predictions by BLAST

		Predicted bacterial hosts							
		S. aureus	***K. pneumoniae***	***A. baumannii***	***P. aeruginosa***	***E. coli***	***S. enterica***	***C. difficile***	***No prediction found***
Actual bacterial hosts	***S. aureus***	47	3	1	0	8	2	9	0
	K. pneumoniae	0	131	7	4	25	7	2	0
	A. baumannii	1	5	45	2	11	0	0	0
	P. aeruginosa	1	33	6	58	15	3	0	1
	E. coli	0	77	23	8	190	26	0	0
	S. enterica	0	4	1	5	40	65	0	0
	C. difficile	1	1	4	0	0	0	15	0

Supplementary Table S6: Hyperparameter values for nested 4-fold cross-validation. Values of different hyperparameters that were considered during nested 4-fold cross-validation to evaluate the performance of the chosen machine learning models: a Linear Discriminant Analysis (LDA) model, a Logistic Regression (LR) model, a Random Forests (RF) model and a Gradient Boosting (GB) model.

**Model**	**Hyperparameter(s)**	**Possible values**
LDA	/	/
LR	C	0.1, 1, 10, 100, 1000
RF	Number of estimators	10, 100, 500
	Percentage of features to consider in looking for the best split	‘auto’, 0.1, 0.25, 0.5
GB	Number of estimators	10, 100, 500

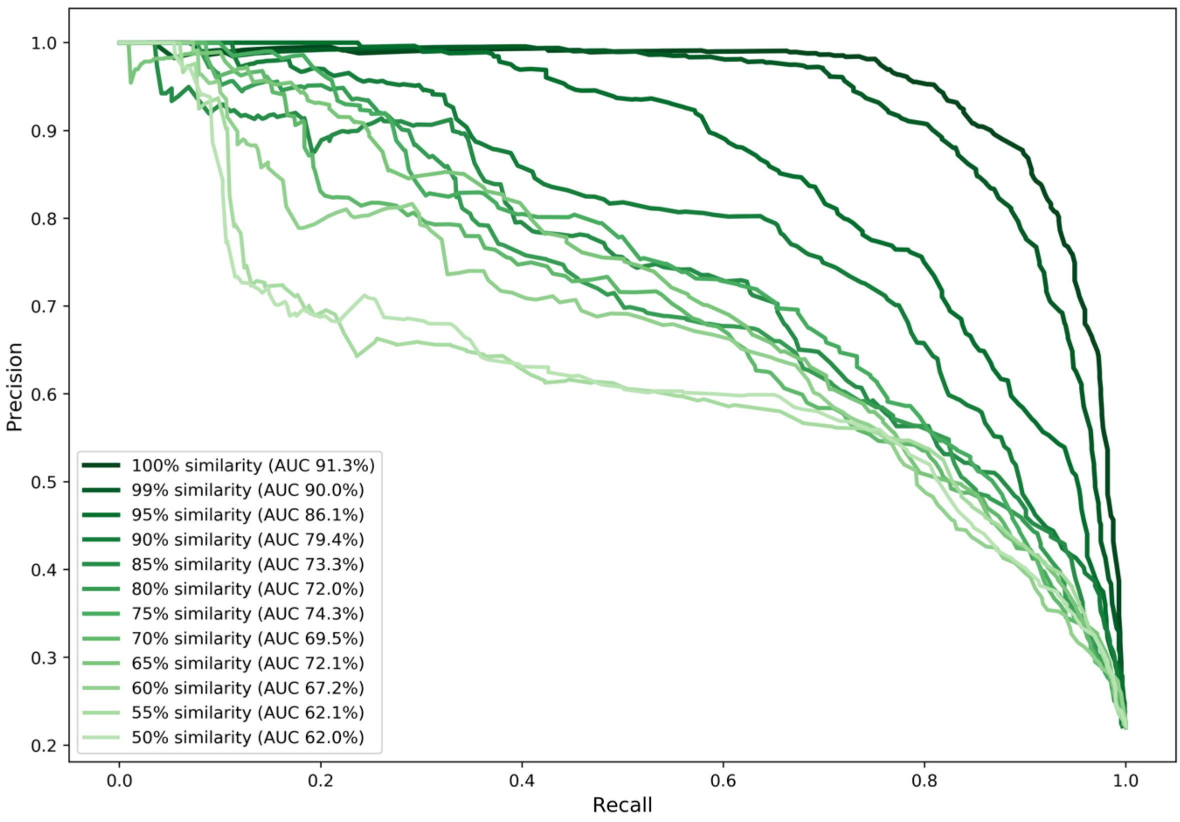

Figure S1: Cross-validated Precision-Recall (PR) curves and Area Under the Curve (AUC) of the best-performing predictive model (RF) trained solely on the N-terminal part of each RBP sequence, across different thresholds for sequence similarity. Grouped, nested 4-fold cross-validation was performed to tune the hyperparameters in the inner loop and compute weighted averaged precision and recall over all classes in the outer loop. This was repeated for different thresholds of sequence similarity in the dataset that controlled the grouping in the cross-validation (i.e. the lower the threshold, the more sequences were grouped into the same fold making test set predictions more difficult). In addition to plotting the PR curves, the AUC was computed as well (see legend).

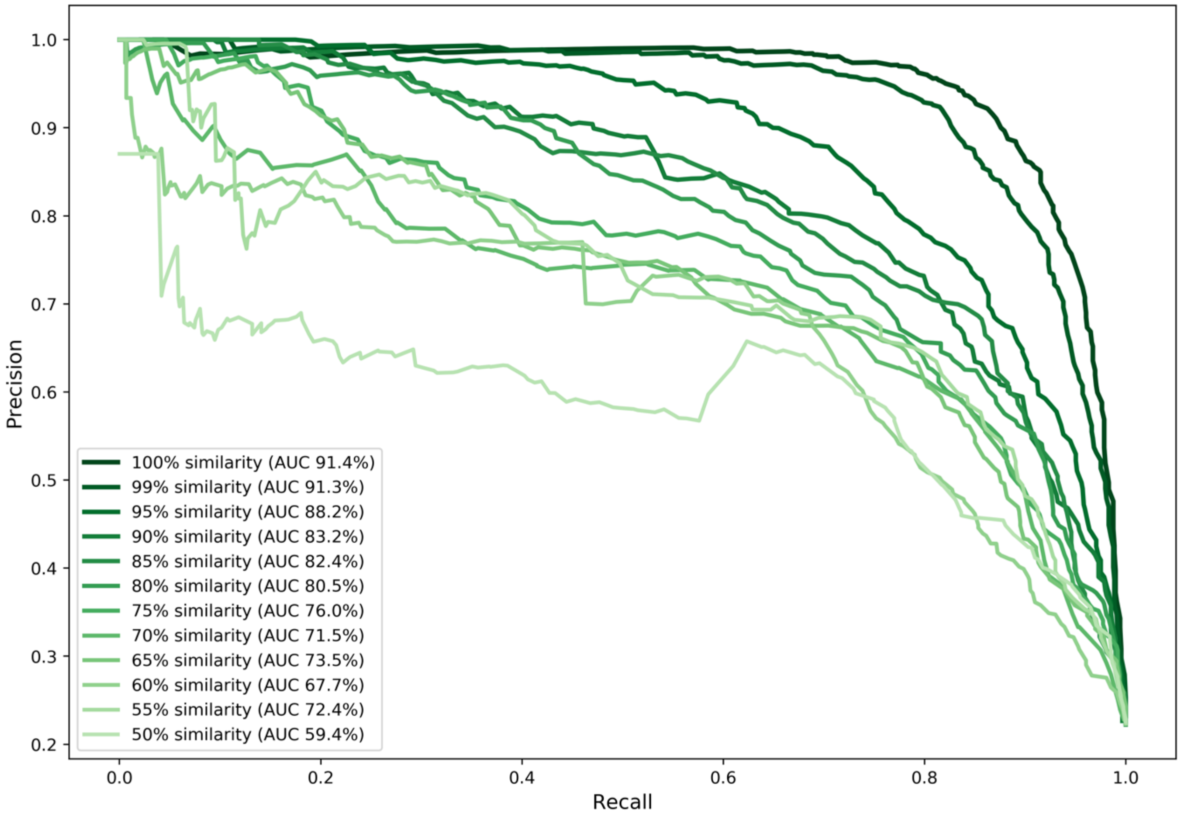

Figure S2: Cross-validated Precision-Recall (PR) curves and Area Under the Curve (AUC) of the best-performing predictive model (RF) trained solely on the C-terminal part of each RBP sequence, across different thresholds for sequence similarity. Grouped, nested 4-fold cross-validation was performed to tune the hyperparameters in the inner loop and compute weighted averaged precision and recall over all classes in the outer loop. This was repeated for different thresholds of sequence similarity in the dataset that controlled the grouping in the cross-validation (i.e. the lower the threshold, the more sequences were grouped into the same fold making test set predictions more difficult). In addition to plotting the PR curves, the AUC was computed as well (see legend).
